# Effects of mobile health interventions on health‐related outcomes in older adults with type 2 diabetes: A systematic review and meta‐analysis

**DOI:** 10.1111/1753-0407.13346

**Published:** 2023-01-17

**Authors:** Jovin Jie Ning Lee, Alia Abdul Aziz, Sok‐Teng Chan, Raja Syazwani Farhanah binti Raja Abdul Sahrizan, Angeline Ying Ying Ooi, Yi‐Ting Teh, Usman Iqbal, Noor Azina Ismail, Aimin Yang, Jingli Yang, Daniel Boon Loong Teh, Lee‐Ling Lim

**Affiliations:** ^1^ Bia‐Echo Asia Center for Reproductive Longevity & Equality (ACRLE), Yong Loo Lin School of Medicine National University of Singapore Singapore; ^2^ Department of Medicine, Faculty of Medicine University of Malaya Kuala Lumpur Malaysia; ^3^ Global Health & Health Security Department, College of Public Health Taipei Medical University Taipei Taiwan; ^4^ Health ICT, Department of Health Canberra Tasmania Australia; ^5^ Department of Economics and Applied Statistics, Faculty of Business and Economics University of Malaya Kuala Lumpur Malaysia; ^6^ Department of Medicine and Therapeutics The Chinese University of Hong Kong Hong Kong China; ^7^ College of Earth and Environmental Sciences Lanzhou University Lanzhou China; ^8^ School of Public Health and Social Work Queensland University of Technology Brisbane Queensland Australia; ^9^ Department of Ophthalmology, Yong Loo Lin School of Medicine National University of Singapore Singapore; ^10^ Department of Anatomy, Yong Loo Lin School of Medicine National University of Singapore Singapore; ^11^ Neurobiology Programme, Life Science Institute National University of Singapore Singapore; ^12^ Asia Diabetes Foundation Hong Kong China

**Keywords:** digital health intervention, older adults, type 2 diabetes mellitus, 数字健康干预, 老年人, 2型糖尿病

## Abstract

**Background:**

Type 2 diabetes mellitus (T2DM) is a chronic metabolic condition that is associated with multiple comorbidities. Apart from pharmacological approaches, patient self‐management remains the gold standard of care for diabetes. Improving patients' self‐management among the elderly with mobile health (mHealth) interventions is critical, especially in times of the COVID‐19 pandemic. However, the extent of mHealth efficacy in managing T2DM in the older population remains unknown. Hence, the present review examined the effectiveness of mHealth interventions on cardiometabolic outcomes in older adults with T2DM.

**Methods:**

A systematic search from the inception till May 31, 2021, in the MEDLINE, Embase, and PubMed databases was conducted, and 16 randomized controlled trials were included in the analysis.

**Results:**

The results showed significant benefits on glycosylated hemoglobin (HbA1c) (mean difference −0.24%; 95% confidence interval [CI]: −0.44, −0.05; *p* = 0.01), postprandial blood glucose (−2.91 mmol/L; 95% CI: −4.78, −1.03; *p* = 0.002), and triglycerides (−0.09 mmol/L; 95% CI: −0.17, −0.02; *p* = 0.010), but not on low‐density lipoprotein cholesterol (−0.06 mmol/L; 95% CI: −0.14, 0.02; *p* = 0.170), high‐density lipoprotein cholesterol (0.05 mmol/L; 95% CI: −0.03, 0.13; *p* = 0.220), and blood pressure (systolic blood pressure −0.82 mm Hg; 95% CI: −4.65, 3.00; *p* = 0.670; diastolic blood pressure −1.71 mmHg; 95% CI: −3.71, 0.29; *p* = 0.090).

**Conclusions:**

Among older adults with T2DM, mHealth interventions were associated with improved cardiometabolic outcomes versus usual care. Its efficacy can be improved in the future as the current stage of mHealth development is at its infancy. Addressing barriers such as technological frustrations may help strategize approaches to further increase the uptake and efficacy of mHealth interventions among older adults with T2DM.

## Graphical abstract

**Highlights**

- Mobile health (mHealth) is an emerging forefront in patient‐centric care.
- Efficacy of mHealth interventions on health outcomes among older adults with type 2 diabetes is limited.
- Modest benefits on cardiometabolic outcomes with mHealth interventions have uncovered critical gaps in the field and offered insights to address barriers at hand.

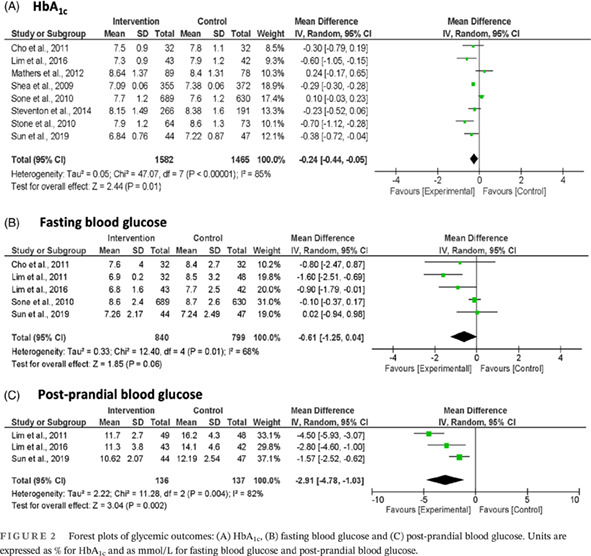

## 摘要

**概述:** 2型糖尿病(T2DM)是一种与多种疾病相关的慢性代谢疾病。除药物治疗外, 患者自我管理仍然是糖尿病治疗的金标准。在COVID‐19大流行期间, 通过移动医疗(mHealth)干预措施改善老年人的自我管理至关重要。然而, 移动医疗在老年人群中治疗2型糖尿病的疗效仍不清楚。因此, 本综述研究了移动健康干预对老年2型糖尿病患者心脏代谢结局的有效性。

**方法:** 从研究开始到2021年5月31日, 对MEDLINE、Embase和PubMed数据库进行了系统检索, 并将16项随机对照试验纳入分析。

**结果:** HbA_1c_(平均差‐0.24%;95% ci: ‐0.44, ‐0.05;p=0.01)、餐后血糖(‐2.91 mmol/L;95%ci:‐4.78, ‐1.03;p=0.002)和甘油三酯(‐0.09mmol/L;95%ci:‐0.17, ‐0.02;p=0.010)明显改善, 而低密度脂蛋白胆固醇(‐0.06 mmol/L;95% ci: ‐0.14, 0.02;p=0.170)、高密度脂蛋白胆固醇(0.05 mmol/L;95% ci: ‐0.03, 0.13;p=0.220)和血压(SBP ‐0.82 mmHg;95% ci: ‐4.65, 3.00;p = 0.670;DBP: ‐1.71 mmHg;95% ci: ‐3.71, 0.29;p = 0.090)并无获益。

**结论:** 在患有2型糖尿病的老年人中, 与常规护理相比, 移动健康干预与改善心脏代谢结果相关。由于当前阶段的移动医疗发展尚处于起步阶段, 其疗效在未来可以得到改进。解决技术等障碍可能有助于策略的制定, 进一步提高患有2型糖尿病的老年人对移动健康干预措施的接受度和有效性。

## 1 INTRODUCTION

Diabetes mellitus is a chronic, metabolic disorder that is characterized by the inability to regulate blood glucose levels.^1^ Worldwide, it is one of the leading causes of morbidity and mortality with a continual rise of incidence rate.^2^ The global estimates of people with diabetes in 2021 approximated 536.6 million, and this number is projected to rise to 783.2 million by 2045, with type 2 diabetes mellitus (T2DM) usually accounting for 90% of all diabetes cases. The prevalence of diabetes also increases with age, with an estimated prevalence of 24% among adults aged 75–79 in 2021 and 24.7% in 2045.^3^

Individual lifestyle, such as a high‐carbohydrate diet, is one of the key contributing factors to the disease development.^4^, ^5^ Diabetes is a lynchpin to a host of cardiometabolic risk factors that would require focused management.^6^ For instance, people with T2DM often experience a host of macro‐ and microvascular complications such as cardiorenal diseases,^7^ with increased odds of deteriorating mental health and quality of life.^8^ As the majority of cardiometabolic risk factors are modifiable, self‐management remains critical in keeping multiple risk factors under control.^4^ Despite the increasing availability of evidence‐based pharmacological and nonpharmacological approaches to tackle diabetes, there are persistent gaps in the delivery of patient‐centered care. This includes a lack of treatment target attainment and use of guideline‐directed medical therapy in people with T2DM, irrespective of national income levels.^9^, ^10^

In a meta‐analysis of 181 randomized clinical trials (RCTs) involving 135 112 patients with T2DM, team‐based care, improved patient–provider communication and continuous self‐management support were the top three quality improvement strategies for reducing multiple cardiometabolic risk factors.^11^ Currently, various mobile health (mHealth) interventions are available to support self‐management while being connected to health care providers. These include, but are not limited to, mHealth applications (apps), glucose sensors/wearables, decision support aids, online educational programs, and telemedicine clinics.^12^ In a meta‐analysis of 13 RCTs involving 1022 patients with T2DM (mean age 45–66 years), the use of the mHealth app was associated with a 0.4% reduction in glycosylated hemoglobin (HbA1c), but not for blood pressure, lipid profile, and body weight.^4^ In this digital age, the use of smartphones, tablet computers, and wearable devices is increasingly prevalent,^13^ and the potential of these mHealth interventions is relevant to older adults aged 65 years and above, given an ever‐increasing aging population, coupled with the fact that this older age group accounts for nearly half of the T2DM population.^14^ Furthermore, the COVID‐19 pandemic presented a unique factor that mandates harnessing such digital platforms more critically^15^ as social distancing becomes an apparent norm. Traditional face‐to‐face clinics are now increasingly replaced with mHealth in this unprecedented time, which potentially adds a cognitive and resource burden on this frail population^16^ to manage T2DM. With this changing norm, it also calls into question the prospect of the use of mHealth interventions in older adults with T2DM.

To our knowledge, there is limited evidence on the effectiveness of mHealth interventions in older adults. Hence, we conducted a systematic review and meta‐analysis of RCTs to examine the effects of digital health interventions on cardiometabolic outcomes in adults with T2DM aged 65 years and older.

## 2 RESEARCH DESIGN AND METHODS

### 2.1 Literature search and screening

**FIGURE 1:**
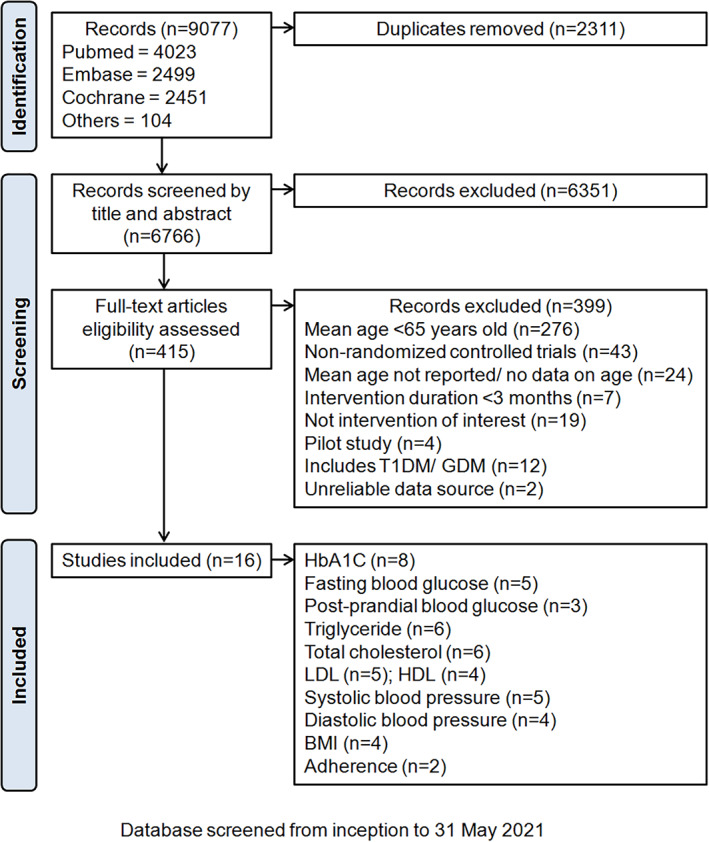
PRISMA diagram

The present meta‐analysis followed the PRISMA (Preferred Reporting Items for Systematic Reviews and Meta‐Analyses) guidelines, and the search was conducted following the PICO (P: patient or problems; I: intervention being considered; C: comparison intervention; O: outcome measurements) framework^17^ with Boolean operators. The protocol was registered on PROSPERO (CRD42020216393). Eligible studies were screened from MEDLINE and Embase using the OVID platform and PubMed from inception till May 31, 2021 (Figure 1). Table 1 shows the search strategy and keywords used (“Diabetes Mellitus, Type 2” and “Telemedicine”). Exploded keywords, MeSH (medical subject headings) terms from MEDLINE, and modified truncation according to the database's format were used.

**TABLE 1:** Search strategy and keywords used

Item (no.)	MeSH search terms
1.	“Diabetes Mellitus, Type 2” [Mesh] OR NIDDM [Title/Abstract] OR T2DM [Title/Abstract] OR T2D [Title/Abstract] OR non insulin depend* [Title/Abstract] OR noninsulin depend* [Title/Abstract] OR noninsulin‐depend* [Title/Abstract] OR non‐insulin‐depend* [Title/Abstract] OR ((type 2 [Title/Abstract] OR type II [Title/Abstract] OR type2 [Title/Abstract] OR typeII [Title/Abstract]) AND diabet* [Title/Abstract]) OR ((late [Title/Abstract] OR adult* [Title/Abstract] OR matur* [Title/Abstract] OR slow [Title/Abstract] OR stabl* [Title/Abstract]) AND onset [Title/Abstract] AND diabet* [Title/Abstract])
2.	“Telemedicine” [Mesh] OR digital health OR Telemedicine OR Mobile Health OR e‐health OR Telehealth OR mobile technolog* OR remote consultation OR e‐mail OR internet OR mobile phone OR telephone OR videoconferencing OR wireless communication OR mobile health OR cell phone* OR telephon* OR mobile OR smartphone* OR smart phone* OR iphone* OR blackberr* OR palmpilot* OR palm pilot* OR android OR pocket pc OR personal digital assistant* OR PDA OR PDAS
3.	Nos. 1 AND 2

Abbreviations: MeSH, medical subject headings; NIDDM, non‐insulin‐dependent diabetes; T2DM, type 2 diabetes mellitus.

The inclusion criteria were RCTs with an interventional duration of 3 months or more, studies reported in English, and older adults with a mean age of 65 years old or more. As this review focused exclusively on T2DM, studies on population groups with type 1 diabetes mellitus (T1DM), gestational diabetes, and a mixture of T2DM with T1DM were excluded. Four reviewers (A.A.A., S.T.C., J.J.N.L., and R.S.F.) independently screened the abstracts and citations, followed by the full‐text articles. Any discrepancies were resolved by a third reviewer (L.L.L.).

### 2.2 Data extraction and analysis

Data from the selected studies were extracted and proofread independently by a reviewer (J.J.N.L.). Blood parameters such as HbA1c, glucose, lipid profile, and blood pressure were excerpted from the studies' terminal point. Other parameters extracted included body mass index (BMI) and adherence rate. The data were analyzed using Revman (version 5.3) to generate the forest and funnel plots. To standardize the units of measures used across studies for analysis, blood glucose and lipid profile parameters are expressed as SI units. As these were continuous variables, the mean difference (MD) with 95% confidence interval (CI) was used. A random‐effects statistical model was selected for all analyses given that true clinical homogeneity could not be assumed.^18^ Heterogeneity was assessed by *I* ^2^ statistics.

To explore the significance of the difference in MDs and the possible influence of confounding factors, we performed subgroup and metaregression analyses on possible sources of heterogeneity,^19^ including country, sex, and duration of follow‐up. We used the “metafor” R package to conduct metaregression in the present study. We assessed the risk of biases of individual studies and the strength of evidence using the GRADE system (Supplementary Table S3).

## 3 RESULTS

### 3.1 Study flow

An initial search of the databases resulted in 9077 RCTs (Figure 1). A total of 16 RCTs were included after the screening process.^17^, ^20^, ^21^, ^22^, ^23^, ^24^, ^25^, ^26^, ^27^, ^28^, ^29^, ^30^, ^31^, ^32^, ^33^, ^34^ The total number of participants was 3257 for the mHealth intervention group and 2947 for the control group in this meta‐analysis. The mHealth interventions included telemonitoring, telecommunication, online education programs, and wearable devices. The duration of intervention ranged from 6 months to 8 years, with a median duration of 1 year. Supplementary Table S1 provides a summary of the included studies. Details on the RCTs' risks of bias and funnel plot analysis are reported in Supplementary Table S3 and Figure S1, respectively.

### 3.2 Glycemic outcomes

**FIGURE 2:**
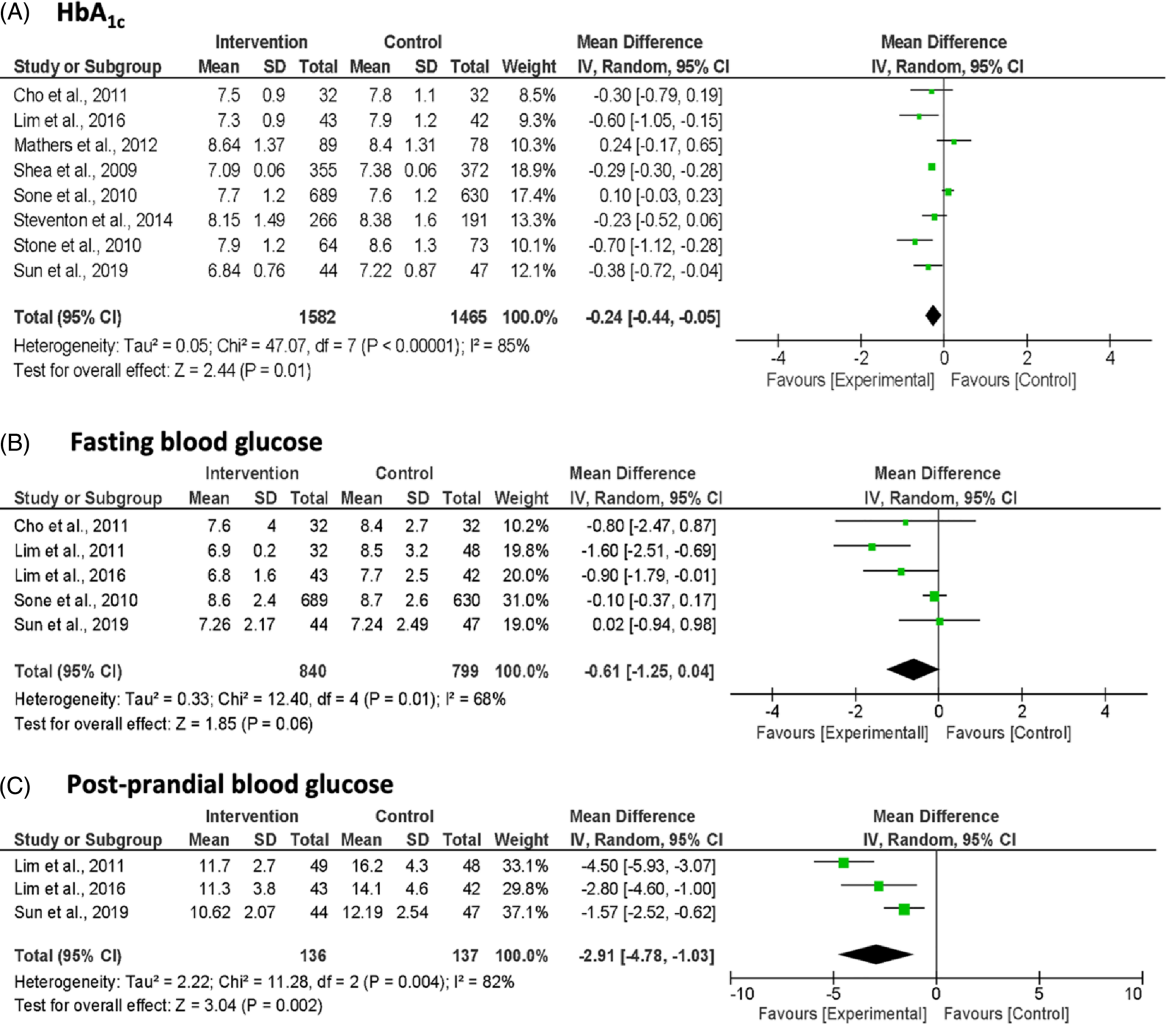
Forest plots of glycemic outcomes: (A) HbA_1c_, (B) fasting blood glucose and (C) post‐prandial blood glucose. Units are expressed as % for HbA_1c_ and as mmol/L for fasting blood glucose and post‐prandial blood glucose.

A total of eight studies reported the glycemic outcomes (Figure 2). The pooled results showed a significant reduction in mean HbA1c of −0.24% (95% CI: −0.44, −0.05; *p* = 0.01; *I* ^2^ = 85%) in the intervention group compared to the usual‐care group. Similarly, postprandial blood glucose from three studies showed a significant reduction in the intervention group over the usual‐care group of −2.91 mmol/L (95% CI: −4.78, −1.03; *p* = 0.002; *I* ^2^ = 82%). However, fasting blood glucose was not significantly different between the intervention and usual‐care groups (MD −0.61 mmol/L [95% CI: −1.25, 0.04; *p* = 0.060; *I* ^2^ = 68%]).

### 3.3 Blood lipid profile

**FIGURE 3:**
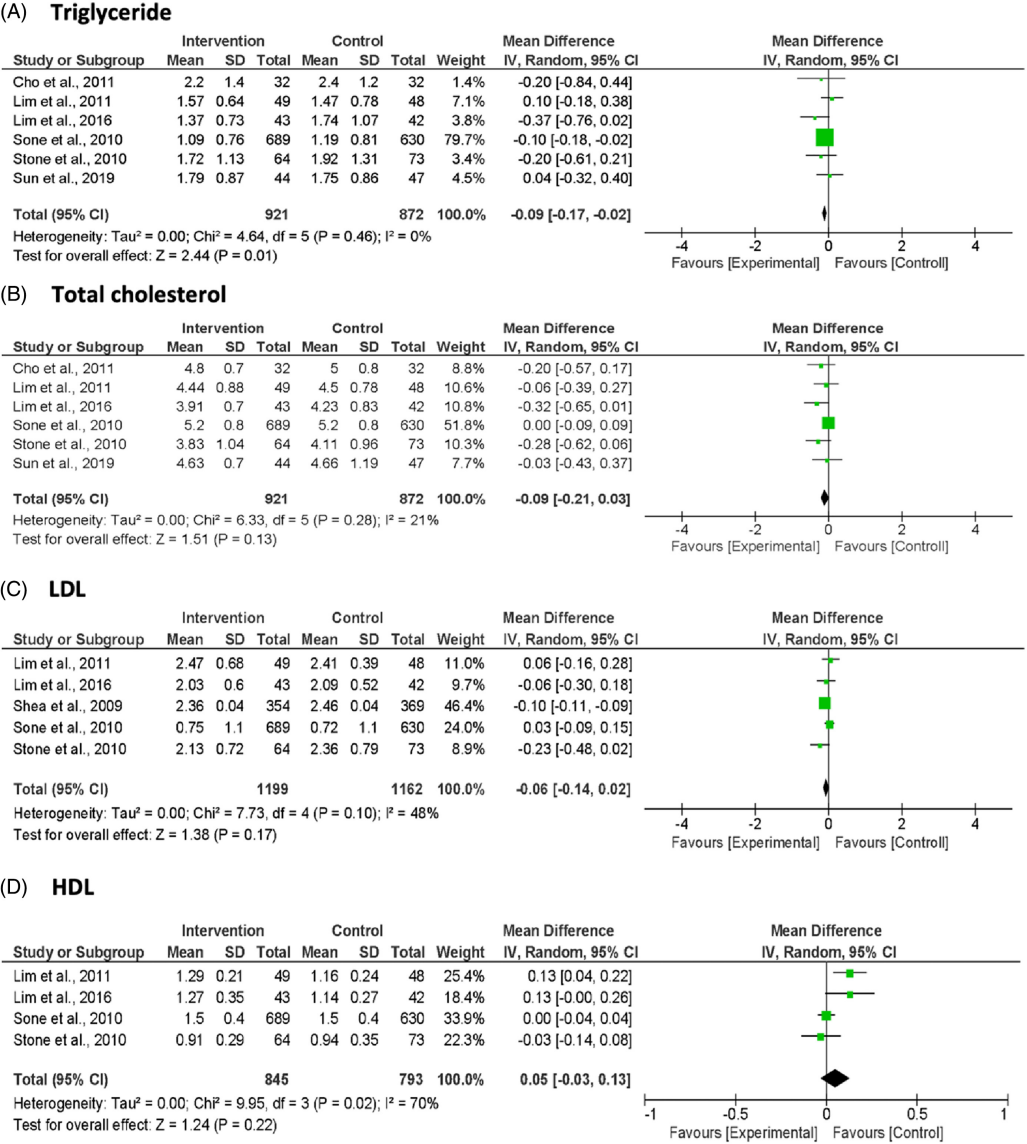
Forest plots of blood lipid profile: (A) Triglyceride, (B) total cholesterol, (C) LDL‐cholesterol, (D) HDL‐cholesterol. Units are expressed as mmol/L.

The pooled MD from six studies showed a significant reduction of triglyceride level by −0.09 mmol/L (95% CI: −0.17, −0.02; *p* = 0.010; *I* ^2^ = 0%) in the intervention group compared to the usual‐care group (Figure 3). Both the total cholesterol and low‐density lipoprotein (LDL) cholesterol levels showed a nonsignificant pooled reduction (total cholesterol: MD −0.09 mmol/L; 95% CI: −0.21, 0.03; *p* = 0.130; *I* ^2^ = 21%; LDL cholesterol: MD −0.06 mmol/L; 95% CI: −0.14, 0.02; *p* = 0.170; *I* ^2^ = 48%), while high‐density lipoprotein (HDL) cholesterol level showed a nonsignificant pooled increase in the intervention group compared with the usual‐care group (MD 0.05 mmol/L; 95% CI: −0.03, 0.13; *p* = 0.220; *I* ^2^ = 70%).

### 3.4 Blood pressure

**FIGURE 4:**
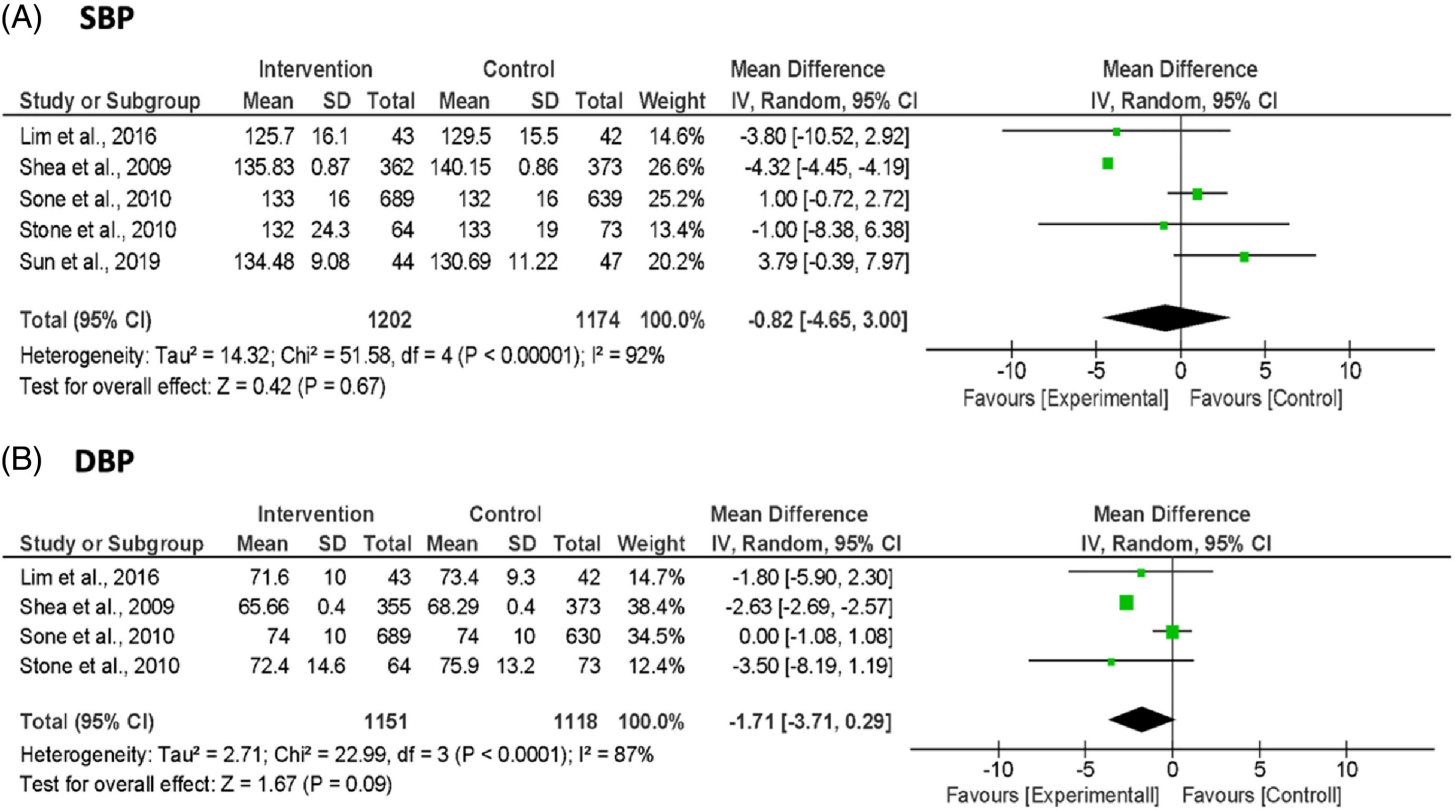
Forest plots of blood pressure: (A) SBP and (B) DBP. Units are expressed as mm Hg.

Five studies reported the outcome on systolic blood pressure (SBP) and four studies on diastolic blood pressure (DBP). The pooled MDs showed a nonsignificant reduction in both SBP (MD −0.82 mm Hg; 95% CI: −4.65, 3.00; *p* = 0.670; *I* ^2^ = 92%) and DBP (MD −1.71 mm Hg; 95% CI: −3.71, 0.29; *p* = 0.090; *I* ^2^ = 87%) (Figure 4).

### 3.5 Other parameters

The intervention groups showed a nonsignificant pooled reduction in BMI of −0.19 kg/m^2^ (95% CI: −0.47, 0.10; *p* = 0.200; *I* ^2^ = 0%) (Supplementary Figure S2). Only two studies independently reported participant adherence rates to interventions, but in different constructs, and could therefore not be included in the forest plot analysis. Lyons et al. (2016) reported better medicine‐taking adherence (odds ratio [OR] 1.54; 95% CI: 1.11, 2.15; *p* = 0.010) and pharmacy‐refill adherence (OR 1.60; 95% CI: 1.14, 2.24; *p* = 0.006) in the intervention group compared with the usual‐care group.^25^ Meanwhile, Wakefield et al. (2011) reported comparable self‐reported medicine‐taking adherence rates between the intervention group receiving telehealth education and monitoring reminders compared to the usual‐care group.^33^

### 3.6 Heterogeneity analysis

**FIGURE 5:**
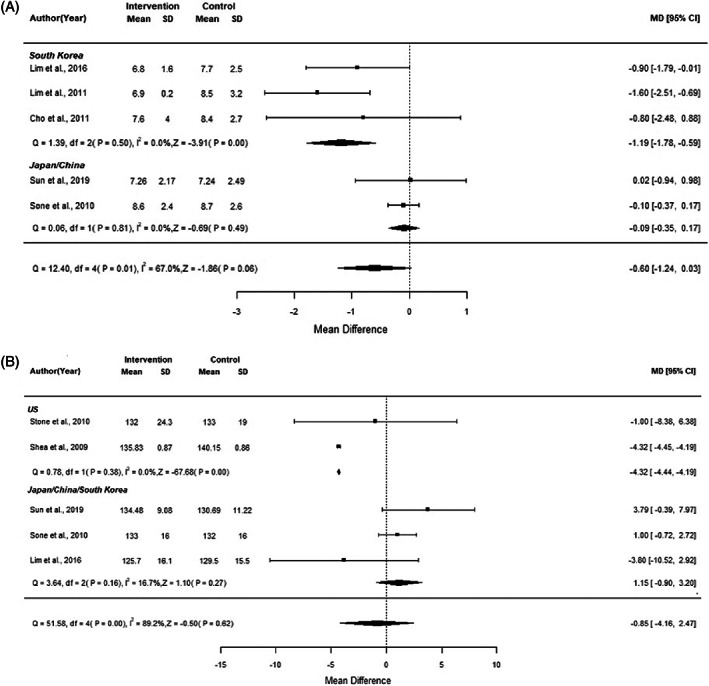
Forest plot of (A) FBG and (B) SBP by Country

We conducted metaregression analyses based on the results of meta‐analyses to determine potential sources of heterogeneity. As shown in Supplementary Table S2, the country might influence the effect of mHealth intervention on FBG and SBP. We observed improved FBG with mHealth intervention in South Korea (MD −1.19; 95% CI: −1.78 to −0.59) (Figure 5). There was also improvement in SBP with mHealth intervention in the USA (MD −4.32; 95% CI: −4.44 to −4.19) (Figure 5).

## 4 DISCUSSION

In this meta‐analysis, we reviewed the evidence on the effects of mHealth interventions in managing T2DM in the older population, age 65 and above. This topic is of paramount significance as the modern age is driven by technology, and mHealth interventions are playing an ever‐growing, pivotal role in health care. It empowers increasing accessibility to health care services, undeterred even in the times of a pandemic.^35^, ^36^ In this respect, COVID‐19 further engenders a tipping point for increased use where socially distanced engagement becomes a new norm, as well as the future to come. Herein, there were variations in forms of mHealth interventions used and the duration of studies. The overall pooled results, however, showed benefits in harnessing them for the betterment of cardiometabolic risk factors in this older population over usual care. We reported improvement in both HbA1c and postprandial blood glucose with mHealth interventions. Likewise, Cui et al., 2016^4^ and Pal et al., 2014^37^ also reported significant positive effects of mHealth interventions on HbA1c. However, several reasons may explain the modest effects of mHealth interventions on this prospect. The impact of mHealth may have waned from a long intervention duration of 3 months or more for reasons such as adherence fatigue.^38^, ^39^

In terms of blood lipid profile, mHealth interventions demonstrated significant reduction in blood triglyceride levels, while effects on other lipid profiles were not statistically significant. Despite T2DM being commonly associated with dyslipidemia,^40^ the relationship between HbA1c and lipid profile appears to remain incongruous. For instance, Alzahrani et al. (2019) reported a significant, positive correlation between HbA1c and triglyceride levels but nonsignificant correlations to total cholesterol, LDL cholesterol, and HDL cholesterol levels.^41^ Meanwhile, Hussain et al. (2017) reported significant, positive correlations between HbA1c, total cholesterol, triglyceride, and LDL cholesterol levels and nonsignificant, negative correlations with HDL cholesterol level.^42^ On the other hand, Begum et al. (2019) found significant correlations between HbA1c, total cholesterol, triglyceride, and HDL cholesterol levels but not with LDL cholesterol.^43^ Regardless, our findings showed support toward an improvement in blood lipid profile, and discrepancies observed in literature may be due to differences in the studied population, interventions given, lifestyle, and environmental factors.

Diabetes often precipitates the onset of hypertension through overlapping etiologies and pathophysiological mechanisms.^44^ Mirroring the small improvement in HbA1c by mHealth interventions, there was no significant improvement in blood pressure. After mHealth interventions, BMI was also not significantly reduced. While several included studies had incorporated exercise and dietary strategies beneficial for weight management, BMI was not the outcome of interest for the majority of the studies. Hence, the small number of studies might be insufficient to substantially demonstrate any effects of the mHealth interventions. Moreover, to a large extent, lifestyle factors contribute to weight management.^45^ However, the degree of adherence to the advised lifestyle regimen was not taken into consideration and could be influenced by the patients' stage of motivation according to the transtheoretical model of change.^46^

Finally, several of the included studies reported on patient adherence to mHealth interventions, particularly telemedicine, with mixed results. This may represent a glaring gap in the literature on mHealth interventions targeting the older population. As highlighted in a scoping review, the participants' age plays a critical role when considering the utility and benefits of mHealth apps, and digital literacy needs to be taken into account as well.^47^ Moreover, approximately 44% of older adults with T2DM experience some form of cognitive dysfunction,^48^ which may hamper their use of mHealth tools. Sustaining the effectiveness of mHealth interventions can be affected in face of nonadherence.^49^ Strategies to ease digital adherence for long‐term cardiometabolic benefits mandate elder‐friendly technology and the joint effort and continuous support from the medical team to train the elderly to become tech‐savvy.^50^

The present meta‐analysis showed that mHealth interventions could improve cardiometabolic risk factors mainly HbA1c, postprandial glucose, and triglycerides in older people with T2DM versus usual care. As highlighted earlier, age may play an important prudent role due to barriers in technological savviness, reduced cognitive capabilities for digital literacy, and accessibility of digital devices. While the use of mHealth among older adults has substantially increased over the years, with a narrowing gap in the digital divide by age, senior age groups have persistently lagged behind in the use of digital resources.^51^ Moreover, the experience of technology frustration, defined by the difficulty to adapt to new technology, especially in older adults, remains a prominent barrier. This encapsulates the lack of motivation and engagement in mHealth interventions that is further aggravated by the lack of face‐to‐face interactions with health professionals to upkeep treatment adherence.^52^ It is therefore important to factor in patients' adherence level when considering the effectiveness of mHealth interventions. Above all, the development of mHealth interventions against chronic diseases is still in the infancy stage given its recency of implementation, even in the context of diabetes.^53^

The main caveats identified in this study are twofold. First, the number of studies included in the meta‐analysis is small. Second, the implementation of mHealth interventions was diverse, and we were not able to analyze by the types of interventions. Third, although there was high heterogeneity for almost all outcomes, our metaregression analysis reported that only “country” might influence the effects of the mHealth interventions on fasting blood glucose and SBP. Last, given that this was a trial‐level meta‐analysis, we were not able to assess the extent of compliance to mHealth intervention and implementation fidelity.

## 5 CONCLUSION

The present meta‐analysis demonstrated the benefits of mHealth interventions on cardiometabolic risk factors compared with usual care for older adults with T2DM, and hence there is utility in incorporating it atop routine care. It can be game‐changing in the delivery of chronic care by facilitating remote disease monitoring and treatment. This is especially useful in situations where face‐to‐face interactions are not feasible or when the older adults have restricted access or limited mobility to attend health care facilities. Nonetheless, the aforementioned barriers have to be addressed in order to further improve the effectiveness of mHealth interventions among older adults with T2DM in real‐word practice.

## AUTHOR CONTRIBUTIONS

Lee‐Ling Lim, Daniel Boon Loong Teh, Usman Iqbal, and Noor Azina Ismail conceptualized the study design. Jovin Jie Ning Lee, Alia Abdul Aziz, Sok‐Teng Chan, and Raja Syazwani Farhanah binti Raja Abdul Sahrizan performed the literature search and appraised the papers. Jovin Jie Ning Lee, Alia Abdul Aziz, and Jingli Yang performed the analysis with support from Lee‐Ling Lim, Aimin, and Noor Azina Ismail. Jovin Jie Ning Lee, Sok‐Teng Chan, Angeline Ying Ying Ooi, and Yi‐Ting Teh wrote the first draft. Lee‐Ling Lim, Aimin Yang, and Daniel Boon Loong Teh finalized the manuscript. All authors revised the manuscript critically for important intellectual content and approved the final version of the paper.

## FUNDING INFORMATION

This work was supported by the Southeast and South Asia and Taiwan Universities (SATU) Joint Research Scheme (ST018‐2020). The funding source did not have any role in the design, interpretation of the study, or the decision to publish the results.

## CONFLICT OF INTEREST

The authors declare that the research was conducted in the absence of any commercial or financial relationships that could be construed as a potential conflict of interest.

## Supporting information


**Data S1.** Supporting Information
