# Supplementary material for: Effects of mobile health interventions on health‐related outcomes in older adults with type 2 diabetes: A systematic review and meta‐analysis
Source: J Diabetes. 2023 Jan 17;15(1):47–57. doi: 10.1111/1753-0407.13346 (PMC9870745; doi:10.1111/1753-0407.13346)
Supplement: Supplementary file 1 — Data S1. Supporting Information [file JDB-15-47-s001.docx]

# Supplementary Materials

**Supplementary Table S1**. Summary of included studies

| **Article** | **Country** | **Age range, n** | **Men,**  **n (%)** | **Intervention** | **Duration of follow-up** | **Outcomes** |
| --- | --- | --- | --- | --- | --- | --- |
| Cho et al.,  2011 (18) | South Korea | T: 36  C: 35 | T: 65.3 ± 9.3  C: 63.1 ± 10.3 | Health-care provider mediated, remote coaching system via a PDA-type glucometer and the Internet | 1 year | HbA_1c_, fasting blood glucose, triglyceride, total cholesterol |
| Dario et al., 2016 (19) | Italy | T: 208  C: 91 | T: 73.05 ± 5.79  C: 73.04 ± 5.28 | Receive telehealth service with a glucometer and gateway for data transmission to a Regional eHealth Center (ReHC) with personalized alarm values of blood glucose | 1 year | Results not in analysis; reported differences within group post-treatment |
| Izquierdo et al., 2010 (20) | US | T: 447  C: 443 | 71.02 ± 7.07  (includes both control and intervention group) | Use of home telemedicine unit to facilitate regular video conference between participants, diabetes nurse manager and dietitian | 2 year | BMI |
| Lim et al., 2011 (21) | South Korea | T: 51  C: 52 | T: 67.2 ± 4.1  C: 68.1 ± 5.5 | Use of individualized medical service, via a PSTN (public switched telephone network)-connected glucometer, where medical instructions were given through the patient’s mobile phone. | 6 months | Fasting blood glucose, post-prandial blood glucose, triglyceride, total cholesterol, LDL, HDL, BMI |
| Lim et al.,  2016 (22) | South Korea | T: 50  C: 50 | T: 65.8 ± 4.7  C: 64.3 ± 5.2 | Use of PSTN (public switched telephone network)-connected glucometer to measure blood glucose level and tailored messages are generated and sent to patients. | 6 months | HbA_1c_, fasting blood glucose, post-prandial blood glucose, triglyceride, total cholesterol, LDL, HDL, SBP. DBP, BMI |
| Lyons et al., 2016 (23) | UK | T: 340  C: 337 | T: 69.9 ± 9.2  C: 69.9 ± 10.1 | Two tele-consults with a pharmacist with written consult summary posted to patients and medicine reminder chart | 6 months | Self-reported non-adherence |
| Mathers et al., 2012 (16) | UK | T: 95  C: 80 | T: 66  C: 62 | Use of PANDAs decision aids with patients in a single consult | 6 months | HbA_1c_ |
| Mons et al., 2013 (24) | Germany | T: 103  C: 101 | T: 68 ^  C: 67 ^ | Receive supportive telephone-based counseling sessions monthly by trained nurses, based on a standardized questionnaire | 1.5 years | Results not in analysis; reported differences within group post-treatment |
| Moriyama et al., 2009 (25) | Japan | T: 42  C: 23 | T: 66.4 ± 9.2  C: 65.2 ± 8.5 | Self-management educational program to improve knowledge of disease and self-care via monthly interviews and biweekly telephone calls. Patients are checked in periodically on adhering goal-setting behaviour | 1 year | Results not in analysis; no SD reported |
| Shea et al., 2009 (26) | US | T: 844  C: 821 | T: 70.8 ± 6.5  C: 70.9 ± 6.8 | Use of home telemedicine unit with nurse case management | 5 year | HbA_1c_, LDL, SBP, DBP |
| Sone et al., 2010 (27) | Japan | T: 1017  C: 1016 | T: 58.5 ± 6.9  C: 58.6 ± 7.0 | Individual counseling for lifestyle factors and treatment adherence, tele-counseling session and diary record for progress | 8 years | HbA_1c_, fasting blood glucose, triglyceride, total cholesterol, LDL, HDL, SBP, DBP, BMI |
| Steventon et al., 2014 (28) | England | T: 300  C: 213 | T: 63.9 ± 13.0  C: 66.2 ± 11.9 | Use of telehealth equipment to send educational messages and remind patients to take physiological measures at frequencies based on patient history | 1 year | HbA_1c_ |
| Stone et al., 2009 (29) | US | T: 64  C: 73 | NS  (1/3 participants are ≥65 years old) | Use of active care management with home telemonitoring device to transmit blood glucose, blood pressure and weight to practitioner nurses for monitoring | 6 months | HbA_1c_, triglyceride, total cholesterol, LDL, HDL, SBP, DBP |
| Sun et al., 2019 (30) | China | T: 44  C: 47 | T: 67.9 ^  C: 68.04 ^ | A mobile phone-based telemedicine, utilizing a mHealth management app to upload glucometer data and provide advice on medication, diet, and exercise. | 6 months | HbA_1c_, fasting blood glucose, post-prandial blood glucose, triglyceride, total cholesterol, SBP |
| Wakefield et al., 2011 (31) | US | T: 93  C: 107 | T: 67.8 ± 10  C: 67.9 ± 9.9 | Blood glucose and pressure are measured daily; Receive guidelines on diabetes and hypertension based on a branching disease management algorithm with educational content | 1 year | Adherence |
| Young et al., 2005 (32) | UK | T: 394  C: 197 | T: 67  C: 67 | Use of pro-active call centre treatment support (PACCTS) where patients are telephoned at the frequency based on their Hba1C to educate lifestyle improvements, motivation for change, medicine adherence and blood glucose management. | 1 year | Results not in analysis; reported % of participants based on specific cut-offs (e.g. HbA_1c_ <7%) |

Footnotes: LDL, low-density lipoprotein cholesterol; SBP, systolic blood pressure; T, treatment group; C, control group; ^, Data reported as median.

**Supplementary Table S2.** Meta-regression of associations between the possible sources of heterogeneity and cardiometabolic outcomes


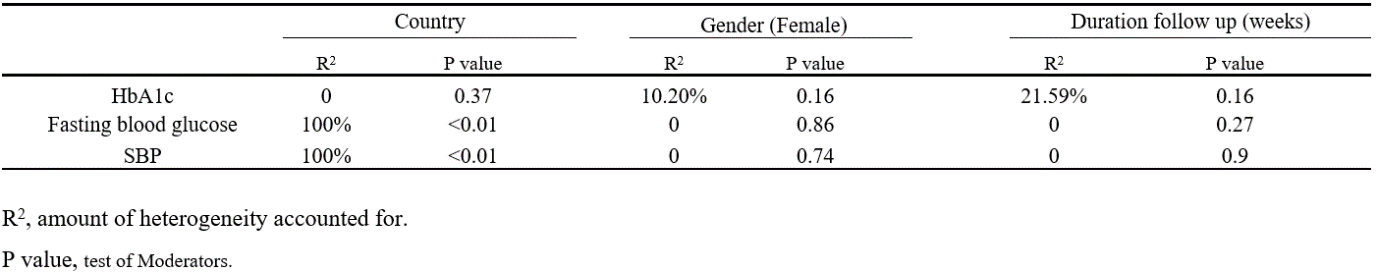


# Supplementary Table S3. Risk of biases of included studies using the GRADE system

**Author(s):**

**Question:** MHealth compared to standard care for managing T2DM cardiometabolic outcomes in older adults

**Setting:**

**Bibliography:** . mhealth for T2DM. Cochrane Database of Systematic Reviews [Year], Issue [Issue].

| **Certainty assessment** | | | | | | | **№ of patients** | | **Effect** | | **Certainty** | **Importance** |
| --- | --- | --- | --- | --- | --- | --- | --- | --- | --- | --- | --- | --- |
| **№ of studies** | **Study design** | **Risk of bias** | **Inconsistency** | **Indirectness** | **Imprecision** | **Other considerations** | **mHealth** | **standard care** | **Relative (95% CI)** | **Absolute (95% CI)** |  |  |
| **Hba1C** | | | | | | | | | | | | |
| 8 | randomised trials | serious^a,b,c^ | serious^d^ | not serious | not serious | dose response gradient | 1582 | 1465 | - | MD **0.24 lower** (0.44 lower to 0.05 lower) | ⨁⨁⨁◯ Moderate | CRITICAL |
| **Total Cholesterol** | | | | | | | | | | | | |
| 6 | randomised trials | serious^a,c^ | not serious | not serious | not serious | none | 921 | 872 | - | MD **0.09 lower** (0.21 lower to 0.03 higher) | ⨁⨁⨁◯ Moderate | IMPORTANT |
| **LDL** | | | | | | | | | | | | |
| 5 | randomised trials | not serious^a^ | not serious | not serious | not serious | none | 1199 | 1162 | - | MD **0.06 lower** (0.14 lower to 0.02 higher) | ⨁⨁⨁⨁ High | IMPORTANT |
| **BMI** | | | | | | | | | | | | |
| 4 | randomised trials | not serious^a^ | not serious | not serious | serious^e^ | none | 994 | 945 | - | MD **0.19 lower** (0.47 lower to 0.1 higher) | ⨁⨁⨁◯ Moderate | CRITICAL |
| **SBP** | | | | | | | | | | | | |
| 5 | randomised trials | not serious^a^ | serious^d^ | not serious | very serious^f^ | none | 1202 | 1174 | - | MD **0.82 lower** (4.65 lower to 3 higher) | ⨁◯◯◯ Very low | CRITICAL |
| **Adherence (follow-up: mean 9 months; assessed with: Scales or questionnaires)** | | | | | | | | | | | | |
| 2 | randomised trials | serious^a,g^ | not serious | not serious | not serious | none | One study reported significantly increased odds in medication and pharmacy-refill adherence. Another study reported no significant differences in medication adherence rate but no data was shown. | | | | ⨁⨁⨁◯ Moderate | CRITICAL |

**CI:** confidence interval; **MD:** mean difference

#### Explanations

a. Blinding was not performed in most studies and understandably due to the nature of intervention

b. Mode of treatment randomization was not reported in one study

c. Cluster RCT design was used in some studies that may lead to inadvertent selection bias

d. There is considerable heterogeneity in the analysis

e. The range of confidence interval is wide.

f. The range of confidence interval is very wide, relative to its effect size, suggesting imprecision.

g. Of the two studies, data was not shown for one of the studies

# Supplementary Figure S1. Funnel plots of study outcomes (a) HbA_1c_, (b) fasting blood glucose, (c) post-prandial blood glucose, (d) triglyceride, (e) total cholesterol, (f) LDL, (g) HDL, (h) SBP, (i) DBP and (j) BMI


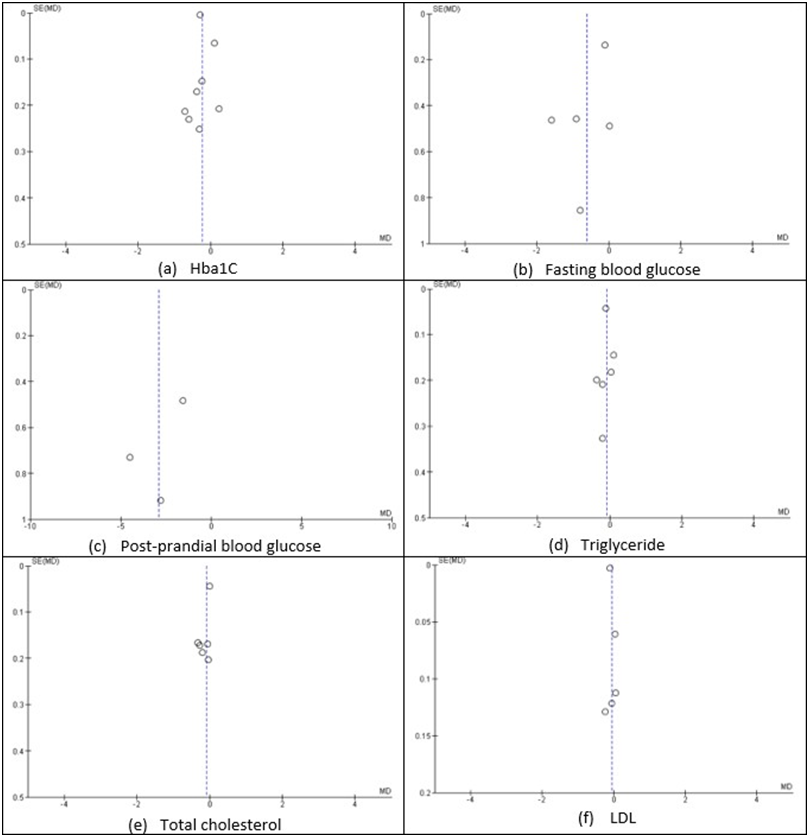


*[Continue below…]*


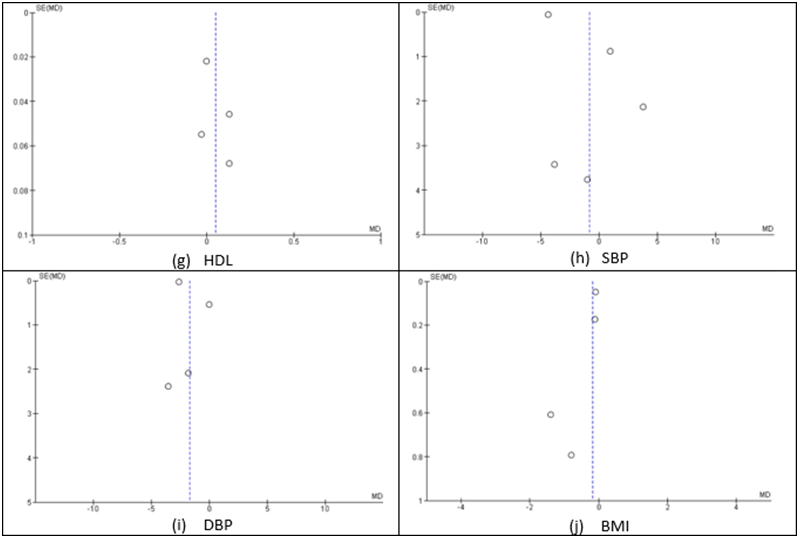


**Supplementary Figure S2.** Forest plot of body mass index (BMI)

*
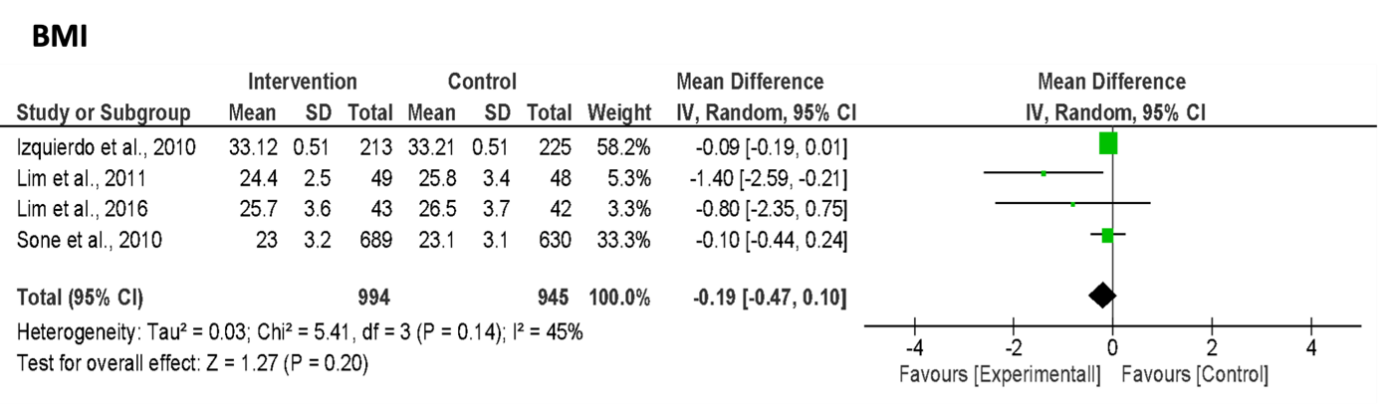
*
